# Variability in Vascular Access Device Use, Dwell Time and Failure: Insights From Routine Nursing Documentation in a Multicentre Cohort Study

**DOI:** 10.1111/jocn.70397

**Published:** 2026-06-18

**Authors:** Ismael Fernández‐Fernández, Enrique Castro‐Sánchez, Miguel Ángel Rodríguez‐Calero, Jesús Bujalance‐Hoyos, José Luís Cobo‐Sánchez, Gaizka Parra‐García, Maria Antonia Cubero‐Pérez, Juan Manuel García‐Garrido, Ian Blanco‐Mavillard

**Affiliations:** ^1^ Espacio de Innovación, Mejora e Investigación Hospital de Manacor Manacor Spain; ^2^ CurES Research Group Health Research Institute of the Balearic Islands (IdISBa) Mallorca Spain; ^3^ Health Protection Research Unit in Healthcare Associated Infection and Antimicrobial Resistance Imperial College London London UK; ^4^ Servicio de Nefrología Hospital Universitario Torrecárdenas Almería Spain; ^5^ Unidad de Terapia Intravenosa Hospital Regional Universitario de Málaga Málaga Spain; ^6^ IBIMA Plataforma Bionand Spain; ^7^ Área de Calidad, Formación, I+D+i de Enfermería Hospital Universitario Marqués de Valdecilla, IDIVAL, UCAV Santander Spain; ^8^ Grupo de Investigación en Inmunopatología Instituto de Investigación Sanitaria Marqués de Valdecilla (IDIVAL) Santander Spain; ^9^ Escuela Universitaria de Enfermería Hospital Mompía Universidad Católica Santa Teresa de Jesús de Ávila (UCAV) Bezana Spain; ^10^ Unidad de Cuidados Intensivos Hospital Son Llàtzer Mallorca Spain; ^11^ Hospital General Universitario Gregorio Marañón Madrid Spain; ^12^ Departamento de Enfermería Universidad Complutense de Madrid Spain; ^13^ Instituto de Investigación Sanitaria Gregorio Marañón (IiSGM) Madrid Spain; ^14^ Equipo de Infusión y Accesos Vasculares Hospital de Inca Mallorca Spain

**Keywords:** catheter failure, nursing, vascular access device, vascular access specialist teams

## Abstract

**Aims:**

To describe and compare institutional patterns of vascular access device use, dwell time, and failure, and to explore their implications for nursing practice.

**Design:**

Retrospective multicentre observational cohort study.

**Methods:**

Routinely collected nursing records of vascular access device insertion and removal were analysed from seven public hospitals over a one‐year period. All devices inserted in hospitalised adults were included, with each device treated as an independent episode. Device failure was defined as removal due to any complication. Device use, dwell time, and failure were summarised by device type and institution. Time‐to‐event analyses were used to describe complication‐free device survival.

**Results:**

More than 200,000 vascular access device episodes were identified, of which ~85,000 had complete data for dwell time and removal reason. Short peripheral intravenous catheters accounted for the majority of devices and reportedly had the shortest dwell time and highest proportion of failure. Medium and long duration devices remained in situ for longer periods and had lower rates of failure, although their use varied substantially between hospitals. Marked inter‐institutional variability was observed in device mix, reported dwell time, complications, and data completeness, with substantial missingness in key variables across hospitals.

**Conclusions:**

Substantial variability exists in vascular access device use, duration, and failure across hospitals. The use of short peripheral intravenous catheters dominates clinical practice despite early failure and limited alignment with expected treatment duration. These findings highlight opportunities to improve device selection and support more consistent, evidence‐based nursing practice, but also underline the need to strengthen institutional data infrastructure and standardise vascular access documentation to enable reliable benchmarking and quality improvement.

**Implications for the Profession and/or Patient Care:**

Optimising vascular access device selection according to anticipated treatment duration may reduce repeated insertions, improve patient comfort, and decrease nursing workload associated with device failure.

**Impact:**

- What problem did the study address?
  ○ Inconsistent use, duration, and documentation of vascular access devices in routine hospital nursing practice.
- What were the main findings?
  ○ Short peripheral intravenous catheters were widely used and failed early, while longer‐duration devices were used less frequently despite lower failure rates. Important gaps in data completeness were also identified across hospitals, particularly for dwell time and removal outcomes.
- Where and whom will the research have an impact?
  ○ The findings are relevant to nurses, nurse leaders, and healthcare organisations involved in vascular access decision‐making and quality improvement.

**Reporting Method:**

This study is reported in accordance with the STROBE guidelines for observational studies.

**Patient or Public Contribution:**

No patient or public contribution.

## What Does This Paper Contribute to the Wider Global Clinical Community?

- Provides large‐scale evidence on real‐world vascular access device use and failure derived from routine nursing documentation.
- Highlights the misalignment between device selection and expected treatment duration in hospital care.
- Supports international efforts to standardise vascular access documentation and improve nursing‐led decision‐making.

## 1 Introduction

Vascular access devices (VADs) are essential for administering fluids, blood products, and medications in hospitalised patients. Approximately 75% of inpatients require at least one VAD during their hospital stay (Alexandrou et al. 2018). Clinicians can use multiple devices to match the clinical indication and expected duration of use, including short peripheral intravenous catheters (PIVCs), peripherally inserted central catheters (PICCs), midlines, central venous catheters (CVCs), and tunnelled or fully implantable devices, among others (Nickel et al. 2024). Given their ubiquity, even small improvements in device selection or maintenance have meaningful implications for safety, resource utilisation, and patient experience (Blanco‐Mavillard et al. 2022; Zingg et al. 2023; Fernandez‐Fernandez et al. 2025).

## 2 Background

Despite their widespread use, VADs are associated with mechanical (e.g., phlebitis, extravasation or infiltration), chemical (drug‐induced phlebitis), and infectious complications (catheter‐related bloodstream infections; CRBSI) (Zingg et al. 2014). These complications frequently arise from suboptimal device selection, improper insertion technique, or inconsistent adherence to evidence‐based protocols for VAD care (Chopra et al. 2015; World Health Organization 2024), underscoring the need for institution‐wide strategies that support appropriate device choice and maintenance.

Appropriate device selection is therefore essential to minimising complications. An improperly sized catheter may cause mechanical issues, while prolonged use of short‐term catheters beyond therapy requirements may increase the risk of chemical or infectious complications (Nickel et al. 2024; World Health Organization 2024; Moureau 2024). Similarly, using devices not recommended for the intended treatment duration may demand repeated insertions throughout the admission, leading to increased patient discomfort and higher healthcare costs (Moureau 2024).

Several interventions and infrastructure can support and improve care surrounding VADs. For example, vascular access specialist teams (VASTs) may help mitigate risks by supporting device selection, insertion and maintenance, and by leading quality‐improvement initiatives. However, these benefits are often limited to the procedures directly performed or supervised, with less evidence on their impact across broader institutional practices (Hallam et al. 2021; Fernandez‐Fernandez et al. 2024; Carr et al. 2018). Further, systematic data collection is essential for evaluating VAD outcomes, monitoring adherence to evidence‐based recommendations, and scaling of quality improvement initiatives through continuous nursing audit (Kwok et al. 2022; Schults et al. 2021). Institutions that maintain structured VAD registries report lower CRBSI rates and better performance metrics (Schults et al. 2021; Instituto Nacional de Estadística 2024).

However, the absence of standardised records is likely to limit reliable benchmarking and impede the assessment of clinical and organisational impact. To address this variability, international experts and clinical guidelines have proposed a minimum dataset for vascular access documentation. This includes key elements such as device type, indication, insertion system, dwell time, complications, and outcomes (Nickel et al. 2024; Moureau 2024; Schults et al. 2021). Despite these recommendations, adoption remains inconsistent, and many institutions continue to rely on fragmented or incomplete records (Nickel et al. 2024; Moureau 2024; Schults et al. 2021).

Understanding institutional practices and outcomes is particularly important in vascular access, where care is predominantly nurse‐led and relies heavily on accurate documentation. Recognising the limited standardisation of vascular access data collection, this study aimed to map institutional datasets and examine variability in device use, dwell time, and complications across hospitals. By doing so, it explores how vascular access activity is captured through routine nursing documentation and how variability in data completeness and structure conditions the extent to which these data can be meaningfully analysed, compared, and used to support benchmarking, quality improvement, and evidence‐based nursing practice.

## 3 The Study

### 3.1 Aims

#### 3.1.1 Main Objective

To describe and compare institutional patterns in VAD use, dwell time, and complication rates across multiple healthcare institutions in Spain, examining how heterogeneous data recording practices contribute to inconsistencies that limit data comparability and interpretation of results.

### 3.2 Objectives

#### 3.2.1 Secondary Objectives

(1) To evaluate the association between the number of full‐time VAST professionals per 100 hospital beds and institutional‐level patterns in device selection, dwell time, and complication rates; (2) To compare VAD types, average dwell time, and reported complications across participating institutions; and (3) To perform time‐to‐event analyses for VAD failure, overall, and stratified per device type and institution.

## 4 Methods

### 4.1 Design

We conducted this retrospective multicentre observational cohort study including all VAD episodes recorded during routine nursing practice from 1 January to 31 December 2023. Seven public hospitals from four autonomous communities (Balearic Islands, Madrid, Cantabria, and Andalusia) participated in the study, representing a mix of tertiary centres and general hospitals, with a combined total of ~4330 inpatient beds. Although all institutions had VASTs, their structure, staffing, and scope varied.

The study was approved by the lead ethics committee of the coordinating centre (the Balearic Islands, code IB 5393/23 PI) and by the local committee of each participating hospital. All datasets were anonymised by the participating institutions prior to transfer and complied with the General Data Protection Regulation (EU 2016/679). This study was reported in accordance with STROBE recommendations for observational cohort studies (Table S1).

### 4.2 Study Setting and Sampling

Twenty‐five hospitals were invited to contribute data and seven provided analysable VAD datasets for the 2023 calendar year. Each VAD episode was treated as an independent unit of analysis, and a unique episode identifier allowed devices to be linked to the corresponding admission where available.

### 4.3 Inclusion and Exclusion Criteria

Inclusion criteria: all records of VADs inserted in hospitalised adults (≥ 18 years) during the study period.

Exclusion criteria were: (1) duplicated records; (2) devices inserted in emergency or outpatient settings without inpatient follow‐up.

### 4.4 Data Sources and Data Collection

A standardised set of ten core variables was agreed by the investigators to maximise comparability across hospitals while accommodating the heterogeneity of existing institutional registries. These variables included: device type; insertion date; removal date; reason for removal; insertion site; department or unit; presence of VAST; patient age; sex; and hospital identifier. The ten core variables were selected as those assumed to be the most consistently recorded across centres and to cover the key domains recommended in international minimum datasets (Schults et al. 2021), while keeping the structure simple and aligned with real‐world registry capabilities.

Catheter failure was defined as catheter removal due to any documented complication. To ensure comparability across institutions, removal reasons reported in local registries were systematically harmonised post hoc into a standardised set of categories including mechanical complications (e.g., extravasation or occlusion), accidental removal, infection‐related events and other device‐related complications. Removal due to end of treatment, patient discharge, or planned replacement of catheter, was classified as complication‐free. The full coding framework, including the mapping of institution‐specific terms to standardised categories is provided in Appendix S1.

To enhance the validity of dwell‐time calculations, predefined thresholds were applied to exclude extreme values unlikely to reflect real clinical practice. Dwell‐time was defined as the number of hours between device insertion and removal, representing the total time the catheter remained in situ. Cut‐off values, established by expert consensus, were ≤ 168 h for short PIVCs, ≤ 720 for micromidlines and midlines, and ≤ 1440 for PICCs and CVCs, reflecting the low probability of longer use in standard care. Values above these thresholds were considered recording errors, such as devices removed in prior admissions but not documented, or removed earlier but registered only at discharge.

### 4.5 Data Analysis

Each vascular access device was treated as an independent unit of analysis, consistent with registry‐based approaches. Multiple devices from the same patient during a single admission were therefore analysed separately, reflecting the structure of routinely collected data.

Descriptive statistics summarised organisational characteristics, patient demographics, device types, dwell times, and complications. Categorical variables were expressed as frequencies and percentages; continuous variables were summarised using means with standard deviations. Group comparisons used chi‐square or Fisher's exact test for categorical data and Student's *t*‐test or Mann–Whitney *U* test for continuous data.

For survival analyses (Kaplan–Meier curves), device failure was defined as removal due to any documented complication (event). Devices removed without any complications (e.g., end of treatment, discharge or planned replacement) were treated as censored observations at the time of removal. Only devices with both valid dwell time and documented reason for removal were included in survival analyses. Devices without a recorded removal reason were excluded from the analyses, as the occurrence of the event could not be determined.

Associations between VAST staffing and outcomes were assessed using Spearman's coefficient. For each device type, relative risks and 95% CIs were computed for each device type by comparing its failure rate with the pooled risk of the remaining devices within the same hospital. A two‐sided *p*‐value < 0.05 was considered significant. Analyses were performed with IBM SPSS Statistics v25 (IBM Corp.).

Analyses of dwell time, complication rates, and survival were conducted using a consistent subset of records with valid and clinically plausible dwell‐time data.

Missing data were handled using a complete‐case approach for each specific analysis. For each analytical component, only records containing the variables required for that analysis were included, as detailed in Appendix S2. No imputation procedures were applied, given the heterogeneity of missingness across centres and variables. As a result, the number of observations varied between analyses, particularly for dwell time and complication‐related outcomes.

### 4.6 Ethical Considerations

Ethical approval and consent to participate: This study was approved by the Research Ethics Committee of the Balearic Islands (code IB 5393/23 PI). Only coded data were used, exported directly by the participating institutions, and no investigators had direct access to patient clinical records. Given the retrospective nature of the study and the use of previously recorded and anonymised data, informed consent was not required. The study was conducted in accordance with the ethical principles of the Declaration of Helsinki and in compliance with the General Data Protection Regulation.

The study used anonymised institutional registry data and contains no individual patient‐identifiable information.

Availability of data and materials: The datasets generated and analysed during the current study contain institutional information from participating hospitals and are not publicly available due to data protection restrictions. Aggregated data supporting the main results are included within the article and its Supporting Information files. Additional anonymised data may be made available from the principal author and/or the corresponding author upon reasonable request and subject to institutional approval.

## 5 Results

### 5.1 Characteristics of the Sample

#### 5.1.1 Hospital Participation and Data Availability

Of the 25 hospitals invited to participate in the study, seven (28%) were able to provide exportable and analysable VAD datasets in the agreed format. Reasons for non‐participation were primarily technical (absence of digital registries, incompatible database structures, or the inability to export electronic health record data). A collaborating researcher at each participating centre extracted the selected variables and transferred anonymised datasets to the study team.

Prior to analysis, dwell‐time outliers exceeding predefined thresholds were excluded as implausible values to ensure the validity of duration‐related results, following the criteria described in the Methods section. Among these, some values were markedly extreme, including PICC records of up to 23,664 h (985 days), PIVC records of 15,905 h (662 days), CVC records of 12,842 h (535 days), midline records of 10,000 h (416 days), and micromidline records of 8124 h (338 days). Appendix S2 and Figure S7 detail data availability across analytical steps, from initial datasets to the final analytical samples. Marked variability in data structure and completeness was observed among the participating hospitals, particularly regarding dwell‐time documentation, which was absent in one hospital and inconsistently reported in others. For example, Hospital G reported dwell time only in intervals, Hospital A provided duration for approximately 7% of records, and Hospital D did not record dwell time at all. These findings reflect heterogeneity in how data are captured and stored across institutions, rather than selective omission. To maximise the completeness and comparability of the analysis, the removal records were prioritised, as they contained more consistent and clinically relevant information, particularly reason for removal, dwell time, and device type. However, other variables available exclusively from insertion records were also incorporated. Missing data for core analytical variables (dwell time, reason for removal and device type) ranged from 5% to 35% across hospitals.

### 5.2 Patient and Device Characteristics

Of the analysed records, 164,707 included demographic information. The mean patient age was 66.2 years (SD ±18.4), with a balanced gender representation (50.3% female) (Table 1). Short PIVCs represented ~90% of all devices. Midlines, micromidlines and PICCs accounted for a small proportion of insertions but showed substantially longer mean dwell times than short PIVCs. Device mix and usage patterns varied between hospitals (Table 2). For example, Hospital D reported the highest proportion of PICC insertions (3.4%), Hospital C the highest proportion of midline insertions (2.3%), whereas Hospital E predominantly used PIVCs (94.2%). Characteristics of the participating centres, including bed capacity and vascular access activity, are summarised in Table 3.

**TABLE 1:** Demographic characteristics of the registered patient population by centre.

Hospital	A	B	C	D	E	F	G
Male (%)	24,306 (46.4%)	6316 (47.7%)	6504 (52.7%)	NA	NA	35,085 (50.4%)	10,343 (52.0%)
Female (%)	28,053 (53.6%)	6942 (52.3%)	5827 (47.3%)	NA	NA	34,476 (49.6%)	9541 (48.0%)
Mean age (SD)	68.7 (17.3)	64.2 (20.2)	64.9 (18.6)	NA	NA	65.4 (18.4)	63.5 (19.3)

Abbreviation: NA, data not available.

**TABLE 2:** Distribution of vascular access device types by centre.

Hospital	A	B	C	D	E	F	G
Short PIVC (%)	46,035 (87.9%)	12,018 (90.6%)	11,261 (91.3%)	18,812 (89.2%)	16,886 (94.2%)	62,764 (90%)	18,375 (93.3%)
Micromidline (%)	67 (0.1%)	169 (1.3%)	0	0	373 (2.1%)	98 (0.1%)	0
Midline (%)	38 (0.1%)	130 (1%)	289 (2.3%)	399 (1.9%)	0	907 (1.3%)	263 (1.3%)
PICC (%)	0	349 (2.6%)	181 (1.5%)	723 (3.4%)	105 (0.6%)	2827 (4.1%)	465 (2.4%)
CVC (%)	1494 (2.9%)	140 (1.1%)	174 (1.4%)	619 (2.9%)	399 (2.2%)	2589 (4%)	418 (2.1%)
Other devices (%)	4725 (9%)	452 (3.4%)	426 (3.5%)	544 (2.6%)	164 (0.9%)	376 (0.5%)	179 (0.9%)
*Devices per 100 hospital beds*							
Short PIVC	5349	1396	1308	2185	1961	729	2134
Micromidline	8	20	0	0	43	11	0
MIDLINE	4	15	34	46	0	105	31
PICC	0	41	21	84	12	328	54
CVC	174	16	20	72	46	301	49
Other devices	549	52	49	63	19	44	21

**TABLE 3:** Characteristics of the participating centres and vascular access activity.

Hospital	A	B	C	D	E	F	G
Number of beds	861	175	224	1000	377	861	832
Specialist teams nurses	1	2	1.5	3.5	1	3	4
Specialist teams nurses assistant	1	0	0	1	0	1	2
Specialist team ratio (nurses) per 100 hospital beds	0.12	1.14	0.67	0.35	0.26	0.35	0.48
Specialist team ratio (all professionals) per 100 hospital beds	0.23	1.14	0.67	0.45	0.26	0.46	0.72
Sample (records)	52,359	13,258	12,331	21,097	17,927	69,561	19,884
Registers per 100 hospital beds	6081	7576	5505	2110	4755	8079	2391

### 5.3 Device Dwell Time and Complications

Short PIVCs had a mean dwell time of 56.4 h (SD ±41.9) and the highest complication rate, with 56.4% removed due to catheter failure, most commonly extravasation (13%). Micromidlines remained in place for a mean of 104.2 h (SD ±111.9), with a 50.4% complication rate (Tables 4 and 5). Midlines and PICCs showed longer dwell times, although with considerable variability between centres. Mean dwell time was 204.7 h (SD ±148.6) for midlines and 278.6 h (SD ±247.7) for PICCs, with corresponding complication rates of 31.0% and 32.9% respectively. Accidental removal was the most frequently reported complication in these devices (6.6% midlines, 5.0% PICCs). CVCs had the longest mean dwell time (224.5 h, SD ±213.8) and the lowest complication rate (30.3%), with accidental removal (4.9%) and extravasation (3.2%) as the most common complications (Tables 4 and 5). Across all device types, devices removed due to catheter failure showed shorter dwell times than those removed without complications (Table 6).

**TABLE 4:** Device dwell time by device type and centre.

Device type	A	B	C	D	E	F	G	Total
Short PIVC	3.7 (20.2)	53.0 (43.0)	57.6 (42.1)	NA	47.5 (36.7)	59.0 (40.2)	70.9 (42.7)	56.4 (41.9)
Micromidline	8.0 (27.7)	96.0 (110.8)	NA	NA	113.2 (116.4)	83.6 (84.0)	NA	104.2 (111.9)
Midline	0.0 (0.0)	170.7 (144.5)	233.1 (167.8)	NA	NA	234.4 (171.1)	156.1 (50.5)	204.7 (148.6)
PICC	NA	266.3 (234.0)	367.1 (346.4)	NA	123.6 (137.0)	329.3 (265.7)	158.0 (55.1)	278.6 (247.7)
CVC	63.5 (168.3)	236.6 (167.9)	297.0 (237.8)	NA	255.9 (248.6)	239.7 (220.5)	148.5 (57.0)	224.5 (213.8)

*Note:* Data are presented as mean dwell time in hours (standard deviation). Dwell time was calculated from insertion to removal of the device, regardless of catheter outcome.

Abbreviation: NA, data not available.

**TABLE 5:** Incidence of catheter failure by device type and centre.

Device type	A	B	C	D	E	F	G	Total
Short PIVC	2923 (91.8%)	3386 (47.5%)	3402 (50.6%)	NA	8809 (54.2%)	16,112 (56.8%)	9276 (57.0%)	43,908 (56.4%)
Micromidline	12 (100%)	51 (52.6%)	NA	NA	177 (48.1%)	18 (51.4%)	NA	258 (50.4%)
Midline	3 (100%)	31 (29.8%)	55 (29.1%)	NA	NA	65 (25.1%)	94 (38.4%)	248 (31.0%)
PICC	NA	60 (23.0%)	14 (12.0%)	NA	34 (32.7%)	208 (27.5%)	222 (56.0%)	538 (32.9%)
CVC	89 (66.9%)	17 (18.4%)	9 (7.4%)	NA	97 (26.4%)	244 (22.8%)	197 (52.7%)	665 (30.3%)

*Note:* Data are presented as number of devices with catheter failure and corresponding percentage. Percentages are calculated using the total number of devices of each type within each centre as the denominator.

Abbreviation: NA, data not available.

**TABLE 6:** Dwell time according to catheter outcome.

Device type	Outcome	Mean hours (SD)	*p* *
Short PIVC	Without complications	59.8 (42.2)	< 0.001
	Catheter failure	52.9 (41.5)	
Micromidline	Without complications	115.7 (112.0)	0.004
	Catheter failure	94.9 (113.9)	
Midline	Without complications	217.0 (146.8)	< 0.001
	Catheter failure	151.8 (114.3)	
PICC	Without complications	287.6 (245.0)	< 0.001
	Catheter failure	232.6 (223.1)	
CVC	Without complications	231.5 (203.3)	< 0.001
	Catheter failure	216.2 (241.4)	

*Note:* Data are presented as mean dwell time in hours (standard deviation). *p* values refer to comparisons between devices removed without complications and those removed due to catheter failure for each device type (Mann–Whitney *U* test). Dwell time was calculated from insertion to removal of the device.

* = comparison between devices removed without complications and those removed due to catheter failure for each device type (Mann‐Whitney U test).

### 5.4 Complications According to Dwell Time

Complication rates varied across dwell‐time categories for all device types (*p* < 0.001). For PIVCs, 61% of failures occurred within the first 24 h compared with 47% at ≥ 144 h. For PICCs, complications declined from 39.8% within the first 5 days to 24% beyond 30 days. Similar trends appeared for micromidlines, midlines, and CVCs (Table S2). Device‐specific 95% confidence intervals are provided in Appendix S3.

### 5.5 Relative Risk of Catheter Failure

Across hospitals, short PIVCs consistently showed the highest relative risk of failure compared with other devices within the same institution (range 1.12–2.82; *p* < 0.001). Midlines, PICCs and CVCs demonstrated significantly lower risks in most hospitals (ranges 0.46–0.67, 0.24–0.61 and 0.14–0.73, respectively). Results for micromidlines were heterogeneous, with only one centre showing a significantly reduced risk (Table S4).

### 5.6 Survival Analysis

Kaplan–Meier curves suggested a gradual decline in complication‐free survival for PIVCs across hospitals. Midlines maintained high, consistent survival. PICCs showed greater variability, partly due to fewer events reported in some centres. CVCs displayed the widest variation, with early failures in some hospitals but extended survival in others (Figures S1–, S6). Device survival refers to the time from insertion until removal due to any complication, representing complication‐free dwell time.

The log‐rank test indicated statistically significant differences in survival distributions between hospitals for all device types (*p* < 0.001). Median complication‐free survival for PIVCs varied from 48.5 h (95% CI 47.0–50.0) to 72.1 h (95% CI 70.0–74.2) across hospitals.

### 5.7 Vascular Access Specialist Team Staffing

No statistically significant associations were observed between the number of full‐time VAST nurses per 100 beds and hospital‐level patterns of device utilisation (Tables 4 and S3) or device‐related outcomes (Table 5). Correlations ranged from weakly positive to weakly negative (*r* from −0.16 to 0.37; all *p* > 0.40). The strongest correlation between staffing and CVC use did not achieve significance (*r* = −0.73; *p* = 0.064), and no consistent pattern was observed across devices.

Similarly, correlations between staffing and device failure rates were uniformly negative, suggesting a trend toward fewer complications in hospitals with higher specialist staffing (*r* from −0.11 to −0.67) but also non‐significant (*p* = 0.145–0.989). These findings should be interpreted cautiously given the limited number of hospitals.

## 6 Discussion

### 6.1 Main Findings

This multicentre analysis of 209,103 VAD records provides, to our knowledge, the first national snapshot of vascular access activity in Spain derived directly from routine institutional electronic documentation. Unlike previous multicentre initiatives with predefined registries (e.g., Flebitis Zero) (Cernuda Martínez et al. 2024; Ferraz‐Torres and Corcuera Martínez 2021), this study harmonised heterogeneous institutional datasets to enable cross‐institutional comparisons. In doing so, it not only offers a realistic view of everyday practice but also demonstrates that variability in documentation systems and data completeness constrains the extent to which vascular access activity can be consistently analysed and compared across institutions.

### 6.2 Interpretation in Relation to Existing Evidence

The patient population was older than that reported in national hospital surveys, reflecting the exclusion of paediatric patients and the typical case‐mix of vascular access users (Instituto Nacional de Estadística 2024). Device utilisation patterns were consistent with previous literature, with short PIVCs representing the vast majority of insertions and contributing disproportionately to complication episodes (Chopra et al. 2015; Hallam et al. 2021).

Device duration and complications aligned broadly with international benchmarks, such as the Vessel Health and Preservation: The Right Approach for Vascular Access (VHP) and the Michigan Appropriateness Guide for Intravenous Catheters (MAGIC) framework (Chopra et al. 2015; Alexandrou et al. 2019). Short PIVCs remained in place for relatively short periods and accounted for most failures (Hallam et al. 2021), whereas midlines and PICCs demonstrated longer dwell time and fewer complications, reinforcing their profile as safer devices when appropriately used (Tripathi et al. 2021; Blanco‐Mavillard et al. 2021). Differences in phlebitis rates between device types were consistent with published evidence and likely reflect clinical indication, insertion site, and drug or medication administered characteristics (Blanco‐Mavillard et al. 2022; Manrique‐Rodriguez et al. 2021).

Comparisons between device types should be attempted cautiously. Vascular access devices are typically selected according to clinical context, treatment characteristics, and patient condition. In the absence of adjustment for case‐mix, treatment indication, or medication properties, differences in observed failure rates may reflect underlying patient selection rather than intrinsic device, team, or institutional performance. Therefore, the comparisons presented in this study should be considered descriptive of real‐world practice patterns rather than evidential of causal differences between devices. From this perspective, the observed variability may be better understood as a reflection of clinical decision‐making processes and organisational practices rather than purely technical differences between devices. This perspective reinforces the importance of considering vascular access as a complex, context‐dependent intervention embedded within routine care (Fernandez‐Fernandez et al. 2025; Castro‐Sánchez et al. 2025).

Although the data in this study come from Spanish hospitals, the challenges observed are unlikely to be unique to this context. These patterns mirror findings from other healthcare systems, where short PIVCs are frequently removed prematurely due to complications such as phlebitis, occlusion, or extravasation, resulting in repeated insertions and increased resource use during hospitalisation (Marsh et al. 2024; Drugeon et al. 2023). International studies have also described substantial variability in reporting practices, reinforcing the need for standardised tools (Fields et al. 2014) and inter‐institutional benchmarking frameworks (Carr et al. 2018). Therefore, although caution is warranted given structural differences between healthcare systems, our findings may be relevant to other settings to improve patient safety and quality of care.

Comparisons with MAGIC recommendations showed shorter‐than‐expected dwell time for both PIVCs and midlines (Chopra et al. 2015). We found that short PIVCs lasted an average of 2.3 days, substantially below the five‐day recommendation. Similarly, midlines averaged 9 days of use, falling short of their 14‐day therapeutic window. While this may reflect real‐world maintenance challenges, interpretation is limited because dwell time is influenced by case‐mix, indication, and documentation practices. Kaplan–Meier curves highlighted early failures, consistent with patterns reported elsewhere. This pattern is consistent with the shorter dwell times observed among devices removed due to complications (Table 6).

The survival analyses should be interpreted in light of the analytical approach used. Because catheter failure was defined as removal due to any documented complication, devices removed without complications were treated as censored observations. However, in routine clinical practice, many devices are removed for reasons unrelated to failure, such as completion of therapy or discharge. As a result, survival estimates reflect complication‐free dwell time rather than total device duration. In addition, the exclusion of devices without a documented removal reason may introduce bias if missingness is not random, potentially influencing survival estimates. Kaplan–Meier curves in this study represent a pragmatic approximation of complication‐free survival based on available documentation, rather than a complete representation of all device trajectories.

Regarding device‐related complications, significant differences were observed among the participating centres. These variations likely reflect differences in documentation practices, reporting culture, and patient or staff mix (Gagliardi et al. 2018; Samore et al. 2004; Choi et al. 2024). While absolute rates varied, the binary classification of failure versus non‐failure appeared more consistent across institutions, suggesting potential utility as a pragmatic outcome measure for benchmarking.

When comparing device duration with 2023 Hospital Morbidity Survey data (Instituto Nacional de Estadística 2024), the mean short PIVC dwell time (2.3 days) (excluding psychiatric admissions) was substantially shorter than the average hospital stay (6.7 days), implying multiple insertions and exposing them to repeated risk of infectious and non‐infectious complications (Zanella et al. 2025). This gap indicated that device selection does not always align with anticipated therapy duration (Chopra et al. 2015).

These findings support a more time‐aligned approach to device selection, consistent with current evidence‐based frameworks (Nickel et al. 2024; Moureau 2024). Reserving PIVCs for short‐term (emergency or < 48‐h) therapy and prioritising midlines or micromidlines for longer treatment courses may reduce unnecessary insertions and improve patient comfort (Fields et al. 2014). Nursing leadership remains central to promoting appropriate device selection and consistent maintenance practices (Morrell 2020).

The lack of association between VAST staffing and institutional outcomes should be interpreted cautiously. With only seven participating hospitals, the statistical power to detect meaningful associations is limited, and the absence of statistically significant findings does not necessarily indicate a lack of effect. However, it is also possible that no true association exists between staffing levels and the outcomes measured in this study. Alternatively, the metrics used may not adequately capture the contribution of VASTs, particularly if their impact is expressed through more complex organisational processes such as decision‐making support, staff training or implementation of evidence‐based practices. Several additional explanations are also plausible, including differences in organisational remit, team maturity, operational scope and local implementation strategies, all of which may influence effectiveness. These findings should therefore be considered hypothesis‐generating rather than definitive. Further research including a larger number of institutions, more detailed characterisation of team structure and function and the use of more sensitive or process‐oriented metrics is needed to clarify the institutional impact of VASTs.

Any institutional cross‐comparison was limited by the substantial variability in documentation practices. Notably, only seven of twenty‐five hospitals approached were able to provide analysable datasets, which in itself reflects a substantial variability in institutional readiness and capacity for documentation and data exploitation. Missingness ranged from 5% to 35%, notably for key variables such as dwell time and reason for removal. This highlights the fragility and heterogeneity of vascular access data systems across institutions. These limitations are not only technical but structural, as they would directly constrain the generation of reliable and useful evidence to evaluate interventions such as VAST implementation and linked outcomes. Strengthening electronic documentation systems and developing standardised vascular‐access registries are key priorities.

Beyond the national context, these findings highlight a global challenge in vascular access management. Internationally, the overuse and premature failure of short PIVCs, heterogeneous documentation, and inconsistent adherence to evidence‐based guidelines remain common in many national healthcare systems (World Health Organization 2024). These inefficiencies affect patient safety, generate operational and economic burdens, and limit opportunities for implementation activities. Coordinated efforts toward standardising documentation practices and integrating quality indicators that enable meaningful benchmarking at both national and international levels are needed.

### 6.3 Strengths and Limitations of the Work

This study has some limitations, mainly related to the heterogeneity of data recording across participating hospitals. A minority of health facilities approached were able to provide usable datasets, primarily due to gaps in data infrastructure and capabilities. This introduces a potential selection bias, as participating institutions may have been more likely to have structured electronic documentation systems, greater organisational support for vascular access programmes, a stronger culture of data recording and audit, or financial headroom. As a result, the observed patterns of device use, documentation completeness and outcomes may not fully reflect practices in hospitals with less developed data systems. This limitation should be considered when interpreting the findings, particularly in relation to generalisability.

A substantial proportion of records received lacked key variables, particularly dwell time and reason for removal, with missingness ranging from 5% to 35%. Although the analytical approach maximised the use of available data by applying complete‐case analyses for each outcome, the implications of missing data should be carefully considered. Missingness was not uniformly distributed across institutions and likely reflected differences in documentation practices rather than random data loss. Therefore, it is plausible that missing data are at least partially systematic, potentially related to local recording habits, workload, or documentation systems.

This has important implications for the interpretation of complication and survival analyses. Incomplete recording removal reasons may lead to underestimation or overestimation of catheter failure rates, depending on whether complications were less likely to be documented or conversely, preferentially recorded. Survival analyses based on documented removal reasons may overrepresent devices with more complete follow‐up, potentially biasing estimates of complication‐free dwell time. These findings should therefore be interpreted as reflecting both clinical practice and the underlying structure and completeness of institutional documentation systems.

In addition, some relevant clinical information, such as therapy indication or type of medication, was not accessible, preventing more detailed adjustment for confounding factors. Similarly, information about VAST composition and functions was available only in aggregated form. We also were not able to adjust our analysis for case‐mix in the different institutions, which may explain the reported over‐ or under‐use of certain devices.

In this study, each vascular access device was treated as an independent analytical unit, consistent with registry‐based approaches. Multiple devices may have referred to the same patient during a single admission spell, and observations may also be correlated within hospitals. This introduces potential statistical dependencies not accounted for in the analysis. As a result, standard errors and confidence intervals may be underestimated, and the precision of estimates should be interpreted with caution. Although this approach reflects the structure of routinely collected data and enables large‐scale analysis, it represents a methodological limitation that should be considered when interpreting the findings.

Despite these limitations, this study provides valuable insight into how vascular access activity is documented and represented within routine hospital information systems. Because the datasets were extracted directly from institutional electronic records, the findings reflect the information that hospitals routinely capture, even if this does not fully correspond to the complete clinical reality. This perspective highlights both the observable patterns in vascular access documentation and the structural gaps that currently limit reliable benchmarking and comparison across institutions. Importantly, the study also demonstrates that constructing large multicentre datasets from routine institutional data is feasible, even in the absence of national registries. This approach can support future efforts to monitor vascular access outcomes, strengthen patient safety initiatives, and advance the development of unified data standards and quality improvement frameworks.

### 6.4 Recommendations for Further Research

Future research should prioritise the development and use of standardised vascular access datasets that enable reliable comparison across institutions. However, the availability of such datasets depends on the existence of adequate institutional data infrastructure. Without robust and interoperable documentation systems, the generation of reliable, comparable data remains limited. Studies incorporating additional clinical variables, such as treatment indication, medication characteristics, and patient case‐mix, would allow more refined adjustment and interpretation of device‐related outcomes. Further work is also needed to explore how organisational factors, including the structure and scope of vascular access specialist teams, influence institutional practice and patient outcomes.

### 6.5 Implications for Policy and Practice

The findings highlight the need for healthcare organisations to better align vascular access device selection with anticipated treatment duration, in order to reduce early device failure and repeated insertions. Strengthening nursing leadership in vascular access decision‐making and promoting the use of medium‐duration devices when clinically appropriate may improve patient comfort and safety. At an institutional level, investment in standardised electronic documentation systems for vascular access is essential to support benchmarking, quality improvement initiatives, and evidence‐based nursing practice.

## 7 Conclusion

A substantial proportion of institutions invited to participate in this study were unable to provide analysable data on vascular access activity, highlighting important limitations in institutional data infrastructure for a routine, high‐volume and potentially harmful area of care. This limitation represents a key structural barrier to reliable benchmarking, evaluation of interventions and the generation of actionable evidence.

This multicentre cohort study reveals substantial variability in vascular access practices across hospitals, as well as in the way these practices are documented. Differences in device selection, dwell time and complication rates were observed, but their interpretation is constrained by heterogeneous documentation systems and variable data completeness across institutions.

Short PIVCs were the predominant device across hospitals, despite short dwell times relative to hospital stay, suggesting that device selection does not consistently align with evidence‐based recommendations regarding anticipated therapy duration. Strengthening the use of medium‐duration devices may help reduce repeated insertions and promote more consistent, evidence‐based practice.

Improving electronic documentation systems and developing standardised vascular‐access registries should be considered a prerequisite for advancing quality of care and supporting evidence‐based nursing practice.

Supporting Information provides additional methodological and analytical detail to support the interpretation of the findings. These include the coding of device removal reasons, data availability across analytical steps, and device‐specific failure rates with corresponding confidence intervals. Supporting Information figures present Kaplan–Meier survival analyses by device type and hospital, as well as a flow diagram illustrating data availability across analytical stages. Supporting Information tables include the STROBE checklist and detailed distributions of catheter outcomes, device use by centre, and risk ratios of catheter failure within centres.

## Author Contributions

**Ismael Fernández‐Fernández:** conceptualization, methodology, formal analysis, writing – original draft, writing – review and editing. **Enrique Castro‐Sánchez:** conceptualization, methodology, writing – review and editing, supervision, writing – review and editing. **Miguel Ángel Rodríguez‐Calero:** data curation, writing – review and editing. **Jesús Bujalance‐Hoyos:** data curation, writing – review and editing. **José Luís Cobo‐Sánchez:** data curation, writing – review and editing. **Gaizka Parra‐García:** data curation, writing – review and editing. **Maria Antonia Cubero‐Pérez:** data curation, writing – review and editing. **Juan Manuel García‐Garrido:** data curation, writing – review and editing. **Ian Blanco‐Mavillard:** conceptualization, methodology, writing – review and editing, supervision.

## Funding

The authors have nothing to report.

## Disclosure

Statistical statement: (a) The authors have checked to ensure that this submission conforms, as applicable, to the Journal's statistical guidelines. (b) The authors affirm that the statistical methods used in the data analyses are appropriate for the study design and context, and that the statistical findings have been correctly implemented and interpreted. (c) The authors agree to take responsibility for ensuring that the choice of statistical approach is appropriate and is conducted and interpreted correctly as a condition of submission to the Journal.

## Conflicts of Interest

The authors declare no conflicts of interest.

## Supporting information


**Appendix S1:** Coding of device removal reasons.

**Appendix S2:** Data availability across analytical steps.

**Appendix S3:** Device‐specific failure rates with 95% confidence intervals.

**Figure S1:** Kaplan‐Meier curves for device survival by type across all centers.

**Figure S2:** Kaplan‐Meier Curves for micromidline by hospital.

**Figure S3:** Kaplan‐Meier Curves for PICC by hospital.

**Figure S4:** Kaplan‐Meier Curves for Short PIVC by hospital.

**Figure S5:** Kaplan‐Meier Curves for Midline by hospital.

**Figure S6:** Kaplan‐Meier Curves for CVC by hospital.

**Figure S7:** Flow diagram of data availability across analytical steps.

**Table S1:** The strengthening of the reporting of observational studies in epidemiology (STROBE) guidelines.

**Table S2:** Distribution of catheter removal outcomes across dwell time intervals by device type.

**Table S3:** Distribution of vascular access devices by centre according to specialist nurse staffing.

**Table S4:** Risk ratio of catheter failure for each device type compared with all other devices within the same centre.

## Data Availability

The data that support the findings of this study are available on request from the corresponding author. The data are not publicly available due to privacy or ethical restrictions.
