# Supplementary material for: Variability in Vascular Access Device Use, Dwell Time and Failure: Insights From Routine Nursing Documentation in a Multicentre Cohort Study
Source: J Clin Nurs. 2026 Jun 18;35(11):4828–38. doi: 10.1111/jocn.70397 (PMC13629244; doi:10.1111/jocn.70397)
Supplement: Supplementary file 1 [file JOCN-35-4828-s006.pdf]

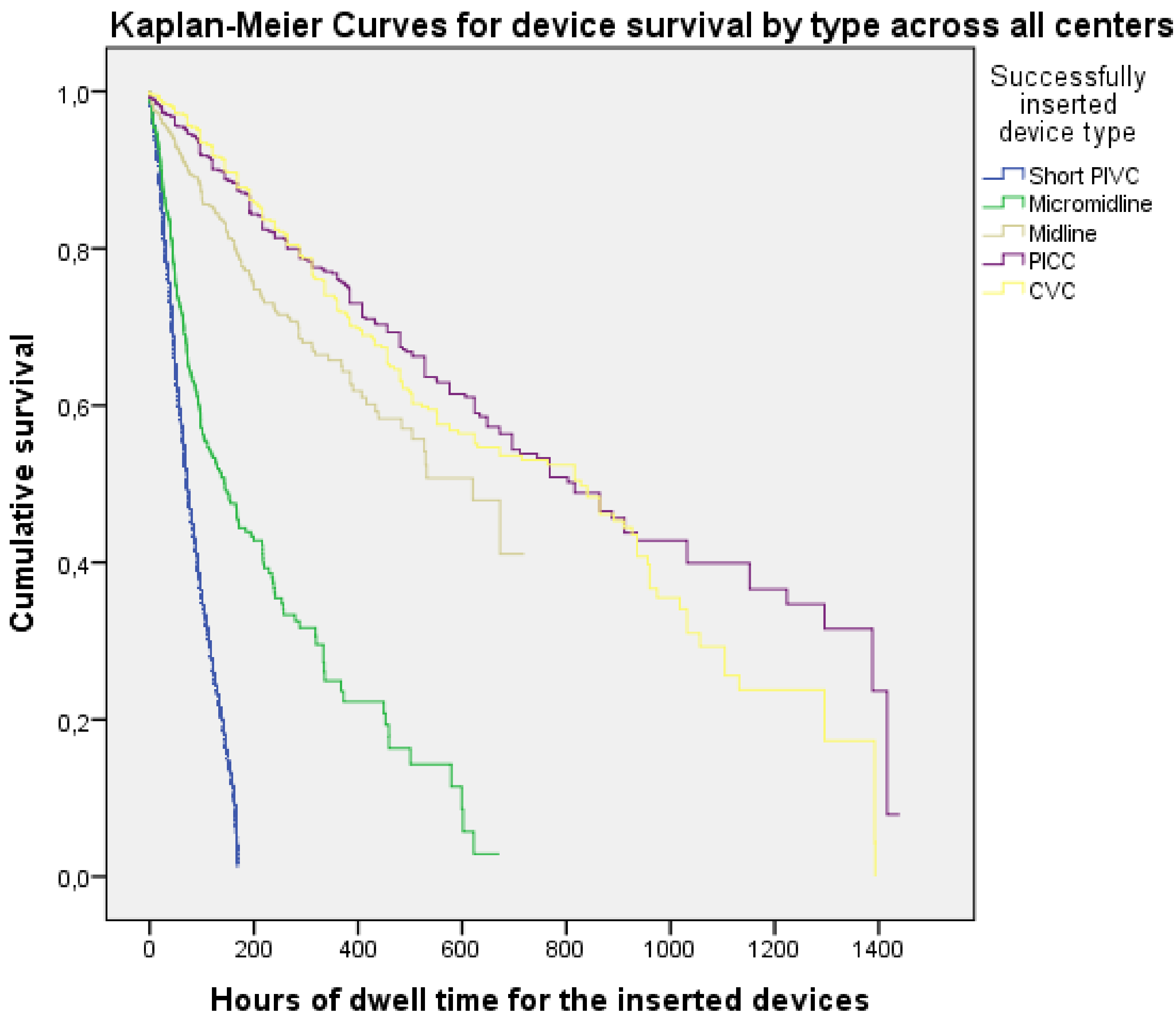

Kaplan-Meier Curves for device survival by type across all centers
Cumulative survival
1,0
0,8
0,6
0,4
0,2
0,0
0
200
400
600
800
1000
1200
1400
Hours of dwell time for the inserted devices
Successfully inserted device type
Short PIVC
Micromidline
Midline
PICC
CVC
