# Supplementary material for: Variability in Vascular Access Device Use, Dwell Time and Failure: Insights From Routine Nursing Documentation in a Multicentre Cohort Study
Source: J Clin Nurs. 2026 Jun 18;35(11):4828–38. doi: 10.1111/jocn.70397 (PMC13629244; doi:10.1111/jocn.70397)
Supplement: Supplementary file 2 [file JOCN-35-4828-s007.pdf]

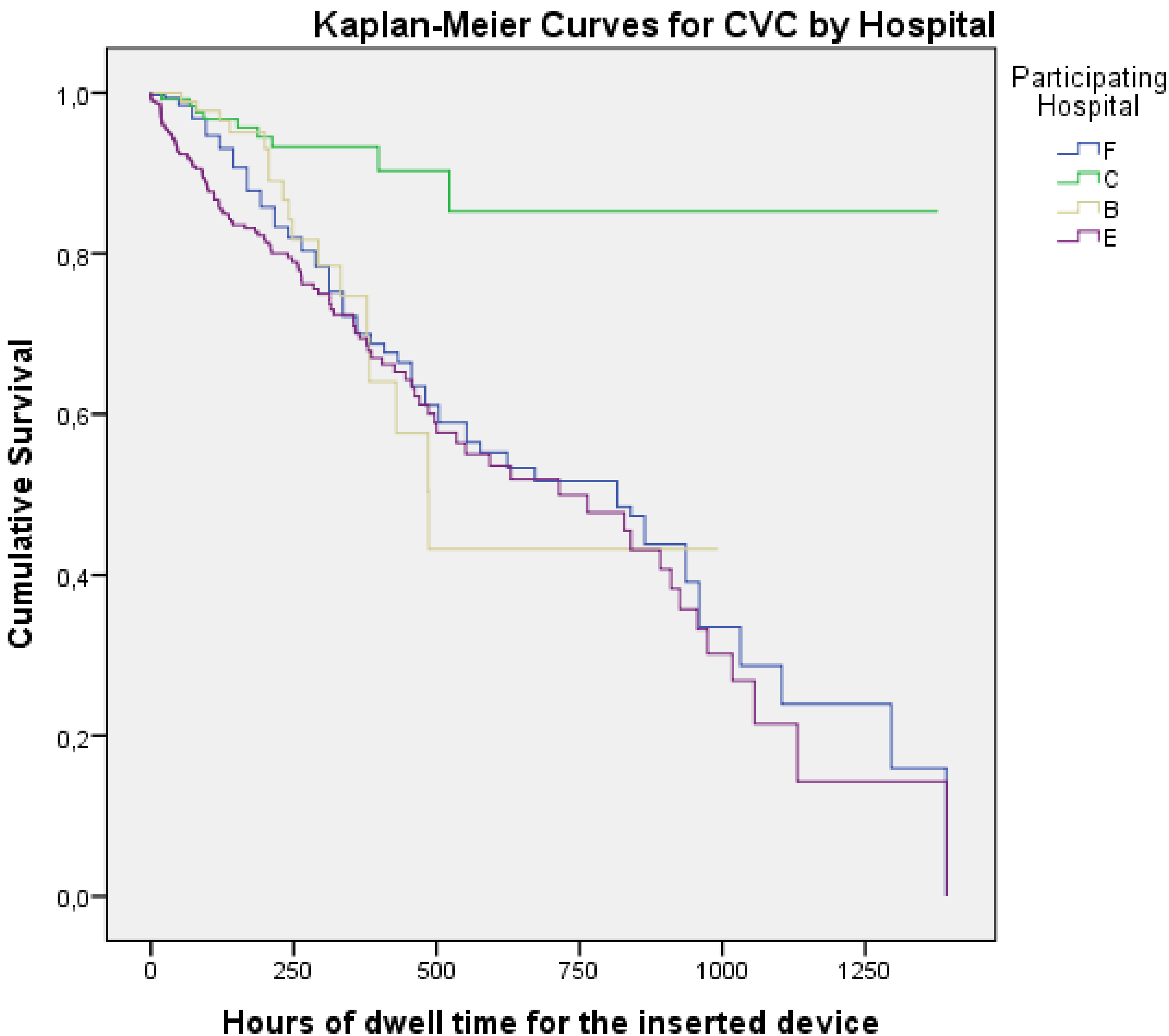
Kaplan-Meier Curves for CVC by Hospital
Cumulative Survival
1,0
0,8
0,6
0,4
0,2
0,0
0
250
500
750
1000
1250
Hours of dwell time for the inserted device
Participating Hospital
F
C
B
E
