# Supplementary material for: Variability in Vascular Access Device Use, Dwell Time and Failure: Insights From Routine Nursing Documentation in a Multicentre Cohort Study
Source: J Clin Nurs. 2026 Jun 18;35(11):4828–38. doi: 10.1111/jocn.70397 (PMC13629244; doi:10.1111/jocn.70397)
Supplement: Supplementary file 7 [file JOCN-35-4828-s001.docx]

**Supplementary table 3.** Distribution of vascular access devices by centre according to specialist nurse staffing.

| HOSPITAL | Nurses Specialist Team ratio / 100 hospital beds | Short PIVC | Micromidline | Midline | PICC | CVC | Other Devices |
| --- | --- | --- | --- | --- | --- | --- | --- |
| A | 0.12 | 46035 (87.9%) | 67 (0.1%) | 38 (0.1%) | 0% | 1494 (2.9%) | 4725 (9%) |
| B | 1.14 | 12018 (90.6%) | 169 (1.3%) | 130 (1%) | 349 (2.6%) | 140 (1.1%) | 452 (3.4%) |
| C | 0.67 | 11261 (91.3%) | 0% | 289 (2.3%) | 181 (1.5%) | 174 (1.4%) | 426 (3.5%) |
| D | 0.35 | 18812 (89.2%) | 0% | 399 (1.9%) | 723 (3.4%) | 619 (2.9%) | 544 (2.6%) |
| E | 0.26 | 16886 (94.2%) | 373 (2.1%) | 0% | 105 (0.6%) | 399 (2.2%) | 164 (0.9%) |
| F | 0.35 | 62764 (90%) | 98 (0.1%) | 907 (1.3%) | 2827 (4.1%) | 2589 (4%) | 376 (0.5%) |
| G | 0.48 | 18375 (93.3%) | 0% | 263 (1.3%) | 465 (2.4%) | 418 (2.1%) | 179 (0.9%) |
| Pearson r with ratio |  | 0.13 | 0.2 | 0.37 | 0.29 | - 0.73 | - 0.16 |
| P - value |  | 0.787 | 0.667 | 0.419 | 0.525 | 0.064 | 0.729 |
