# Supplementary material for: Variability in Vascular Access Device Use, Dwell Time and Failure: Insights From Routine Nursing Documentation in a Multicentre Cohort Study
Source: J Clin Nurs. 2026 Jun 18;35(11):4828–38. doi: 10.1111/jocn.70397 (PMC13629244; doi:10.1111/jocn.70397)
Supplement: Supplementary file 8 [file JOCN-35-4828-s002.docx]

**Supplementary appendix 1**. Coding of device removal reasons.

| Removal Reason | Institution-reported terms |
| --- | --- |
| No complications | Change of lumen/device/site, Change per clinical guidelines, Death, Device from previous episode, Discharge, Follow-up in another facility, No complication recorded, None, Not needed / End of treatment, Planned removal, Planned removal: appropriateness, Planned removal: death, Planned removal: discharge, Planned removal: end of treatment, Planned removal: not needed, Scheduled replacement, Treatment completed |
| Phlebitis | Phlebitis, Phlebitis (PICC), Phlebitis (graded), Suspected phlebitis |
| Extravasation/Infiltration | Extravasation, Infiltration, Leakage of non-vesicant, Leakage of vesicant |
| Pain/Discomfort | Insertion site pain, Non-phlebitis discomfort, Pain, Patient-reported discomfort |
| Occlusion | Blockage, Occlusion, Unmanageable occlusion |
| Accidental removal | Accidental extraction, Accidental removal, Unplanned dislodgement |
| Documented bloodstream infection | CRBSI, Confirmed infection |
| Suspected infection | Other infection-related complications, Signs of infection, Suspected infection, Unconfirmed infection |
| Other (breakage, leakage, etc.) | Catheter breakage, Chemotherapy protocol, Hematoma, Hemorrhage, Inappropriate device, Insertion site dilation, Insertion site leakage, Mechanical malfunction, Not applicable (complication occurred), Pneumothorax, Skin injury, Thrombosis, Venous thrombosis |
