# Supplementary material for: Variability in Vascular Access Device Use, Dwell Time and Failure: Insights From Routine Nursing Documentation in a Multicentre Cohort Study
Source: J Clin Nurs. 2026 Jun 18;35(11):4828–38. doi: 10.1111/jocn.70397 (PMC13629244; doi:10.1111/jocn.70397)
Supplement: Supplementary file 9 [file JOCN-35-4828-s003.docx]

**Supplementary Figure 7**. Flow diagram of data availability across analytical steps

Note Analytical subsets were not derived sequentially. Each analysis used the full dataset (n = 209,103) and excluded only records lacking the specific variables required.
