# Supplementary material for: Variability in Vascular Access Device Use, Dwell Time and Failure: Insights From Routine Nursing Documentation in a Multicentre Cohort Study
Source: J Clin Nurs. 2026 Jun 18;35(11):4828–38. doi: 10.1111/jocn.70397 (PMC13629244; doi:10.1111/jocn.70397)
Supplement: Supplementary file 10 [file JOCN-35-4828-s004.docx]

**Supplementary table 2**. Distribution of catheter removal outcomes across dwell time intervals by device type.

| **SHORT PIVC** | **Removal without complication** | **Removal due to catheter failure** | **TOTAL** | **CUMULATIVE TOTAL** | **CHI SQUARE** |
| --- | --- | --- | --- | --- | --- |
| **≤24 HOURS** | **8859 (39%)** | **13660 (61%)** | **22519 (29%)** | **29%** | **P <0.001** |
| **25–48 HOURS** | **6961 (45%)** | **8515 (55%)** | **15476 (20%)** | **49%** |  |
| **49–72 HOURS** | **8190 (43%)** | **10770 (57%)** | **18960 (24%)** | **73%** |  |
| **73–96 HOURS** | **3081 (46%)** | **3678 (54%)** | **6759 (9%)** | **82%** |  |
| **97–120 HOURS** | **2189 (48%)** | **2335 (52%)** | **4524 (6%)** | **88%** |  |
| **121–143 HOURS** | **3555 (47%)** | **3974 (53%)** | **7529 (10%)** | **97%** |  |
| **≥144 HOURS** | **1118 (53%)** | **976 (47%)** | **2094 (3%)** | **100%** |  |
| **TOTAL** | **33953 (44%)** | **43908 (56%)** | **77861** |  |  |
| **MICROMIDLINE** | **Removal without complication** | **Removal due to catheter failure** | **TOTAL** | **CUMULATIVE TOTAL** | **CHI SQUARE** |
| **≤24 HOURS** | **61 (48%)** | **65 (52%)** | **126 (25%)** | **25%** | **P = 0.002** |
| **25–48 HOURS** | **28 (37%)** | **48 (63%)** | **76 (15%)** | **39%** |  |
| **49–72 HOURS** | **23 (36%)** | **41 (64%)** | **64 (13%)** | **52%** |  |
| **73–96 HOURS** | **20 (44%)** | **25 (56%)** | **45 (9%)** | **61%** |  |
| **97–120 HOURS** | **27 (59%)** | **19 (41%)** | **46 (9%)** | **70%** |  |
| **121–143 HOURS** | **19 (66%)** | **10 (34%)** | **29 (6%)** | **75%** |  |
| **≥144 HOURS** | **76 (60%)** | **50 (40%)** | **126 (25%)** | **100%** |  |
| **TOTAL** | **254 (50%)** | **258 (50%)** | **512** |  |  |
| **MIDLINE** | **Removal without complication** | **Removal due to catheter failure** | **TOTAL** | **CUMULATIVE TOTAL** | **CHI SQUARE** |
| **≤24 HOURS** | **27 (47%)** | **30 (53%)** | **57 (7%)** | **7%** | **P <0.001** |
| **25–48 HOURS** | **9 (43%)** | **12 (57%)** | **21 (3%)** | **10%** |  |
| **49–72 HOURS** | **30 (54%)** | **26 (46%)** | **56 (7%)** | **17%** |  |
| **73–96 HOURS** | **29 (73%)** | **11 (27%)** | **40 (5%)** | **22%** |  |
| **97–120 HOURS** | **20 (63%)** | **12 (37%)** | **32 (4%)** | **26%** |  |
| **121–143 HOURS** | **92 (68%)** | **43 (32%)** | **135 (17%)** | **43%** |  |
| **≥144 HOURS** | **345 (75%)** | **114 (25%)** | **456 (57%)** | **100%** |  |
| **TOTAL** | **552 (69%)** | **248 (31%)** | **797** |  |  |
| **MIDLINE** | **Removal without complication** | **Removal due to catheter failure** | **TOTAL** | **CUMULATIVE TOTAL** | **CHI SQUARE** |
| **≤5 days** | **115 (55,8%)** | **91 (44,2%)** | **206 (25,7%)** | **25,7%** | **P <0.001** |
| **6–10 days** | **275 (68,4%)** | **127 (31,6%)** | **402 (50,2%)** | **75,9%** |  |
| **11–15 days** | **85 (85,9%)** | **14 (14,1%)** | **99 (12,4%)** | **88,3%** |  |
| **16–20 days** | **35 (80%)** | **9 (20%)** | **44 (5%)** | **93,3%** |  |
| **21–25 days** | **26 (84%)** | **5 (16%)** | **31 (3,9%)** | **97,2%** |  |
| **26–30 days** | **16 (89%)** | **2 (11%)** | **18 (2,2%)** | **100%** |  |
| **>30 days** | **0** | **0** | **0** |  |  |
| **TOTAL** | **552 (69%)** | **248 (31%)** | **800** |  |  |
| **PICC** | **Removal without complication** | **Removal due to catheter failure** | **TOTAL** | **CUMULATIVE TOTAL** | **CHI SQUARE** |
| **≤5 days** | **229 (59,6%)** | **155 (40,4%)** | **384 (23,5%)** | **23,5%** | **P <0.001** |
| **6–10 days** | **426 (62,6%)** | **254 (37,4%)** | **680 (41,5%)** | **65%** |  |
| **11–15 days** | **166 (83,8%)** | **32 (16,2%)** | **198 (12,1%)** | **77,1%** |  |
| **16–20 days** | **101 (72,7%)** | **38 (27,3%)** | **139 (8,5%)** | **85,6%** |  |
| **21–25 days** | **63 (77,8%)** | **18 (22,2%)** | **81 (4,9%)** | **90,5%** |  |
| **26–30 days** | **37 (68,5%)** | **17 (31,5%)** | **54 (3,3%)** | **93,8%** |  |
| **>30 days** | **76 (76%)** | **24 (24%)** | **100 (6,1%)** | **100%** |  |
| **TOTAL** | **1098 (67,1%)** | **538 (32,9%)** | **1636** |  |  |
| **CVC** | **Removal without complication** | **Removal with complication** | **TOTAL** | **CUMULATIVE TOTAL** | **CHI SQUARE** |
| **≤5 days** | **497 (67,5%)** | **239 (32,5%)** | **736 (33,5%)** | **33,5%** | **P <0.001** |
| **6–10 days** | **533 (67,5%)** | **257 (32,5%)** | **790 (36%)** | **69,5%** |  |
| **11–15 days** | **232 (77,3%)** | **68 (22,7%)** | **300 (13,7%)** | **83,2%** |  |
| **16–20 days** | **130 (77,4%)** | **38 (22,7%)** | **168 (7,6%)** | **90,8%** |  |
| **21–25 days** | **43 (68,3%)** | **20 (31,7%)** | **63 (2,9%)** | **93,7%** |  |
| **26–30 days** | **41 (85,4%)** | **7 (14,6%)** | **48 (2,2%)** | **95,9%** |  |
| **>30 days** | **53 (59,6%)** | **36 (40,4%)** | **89 (4%)** | **100,00%** |  |
| **TOTAL** | **1529 (69,7%)** | **665 (30,3%)** | **2194** |  |  |
