# Supplementary material for: Variability in Vascular Access Device Use, Dwell Time and Failure: Insights From Routine Nursing Documentation in a Multicentre Cohort Study
Source: J Clin Nurs. 2026 Jun 18;35(11):4828–38. doi: 10.1111/jocn.70397 (PMC13629244; doi:10.1111/jocn.70397)
Supplement: Supplementary file 11 [file JOCN-35-4828-s005.docx]

**Supplementary table 4.** Risk ratio of catheter failure for each device type compared with all other devices within the same centre.

| Hospital | Relative Risk Catheter Failure (CI 95%) | P |
| --- | --- | --- |
| Short PIVC | | |
| A | 1.31 (1.18-1.45) | <0.001 |
| B | 1.66 (1.45-1.90) | <0.001 |
| C | 2.82 (2.30-3.46) | <0.001 |
| D | NA |  |
| E | 1.46 (1.34-1.6) | <0.001 |
| F | 2.25 (2.09-2.42) | <0.001 |
| G | 1.12 (1.06-1.20) | <0.001 |
| Micromidline | | |
| A | 1.10 (1.08-1.11) | 0.271 |
| B | 1.14 (0.94-1.38) | 0.2 |
| C | NA |  |
| D | NA |  |
| E | 0.89 (0.8-1) | 0.041 |
| F | 0.94 (0.68-1.30) | 0.7 |
| G | NA |  |
| Midline | | |
| A | 1.10 (1.08-1.11) | 0.582 |
| B | 0.64 (0.47-0.86) | 0.001 |
| C | 0.59 (0.47-0.74) | <0.001 |
| D | NA |  |
| E | NA |  |
| F | 0.46 (0.37-0.56) | <0.001 |
| G | 0.67 (0.57-0.79) | <0.001 |
| PICC | | |
| A | NA |  |
| B | 0.49 (0.39-0.61) | <0.001 |
| C | 0.24 (0.15-0.39) | <0.001 |
| D | NA |  |
| E | 0.61 (0.46-0.80) | <0.001 |
| F | 0.49 (0.44-0.55) | <0.001 |
| G | 0.98 (0.90-1.07) | 0.755 |
| CVC | | |
| A | 0.73 (0.64-0.82) | <0.001 |
| B | 0.38 (0.25-0.59) | <0.001 |
| C | 0.14 (0.07-0.26) | <0.001 |
| D | NA |  |
| E | 0.51 (0.44-0.60) | <0.001 |
| F | 0.41 (0.36-0.45) | <0.001 |
| G | 0.92 (0.84-1.02) | 0.113 |

+ Reference group: All other devices in the same centre.
