# Supplementary material for: Variability in Vascular Access Device Use, Dwell Time and Failure: Insights From Routine Nursing Documentation in a Multicentre Cohort Study
Source: J Clin Nurs. 2026 Jun 18;35(11):4828–38. doi: 10.1111/jocn.70397 (PMC13629244; doi:10.1111/jocn.70397)
Supplement: Supplementary file 12 [file JOCN-35-4828-s008.docx]

**Supplementary appendix 2.** Data availability across analytical steps

A total of 209,103 vascular access device records were extracted from participating hospitals.

Depending on the variables required for each analysis, the number of usable records varied as follows:

| Analytical step | Excluded | Available records | Notes |
| --- | --- | --- | --- |
| Patient characteristics (age, sex) | 41710 missing | **167393** available | Used for demographic analyses |
| Device characteristics (device type) | 2870 missing | **206233** available | Only 1.37% missing device type |
| Device dwell time | 109082 missing or implausible dwell time | **100021** available | Includes 6866 “other device types” |
| Complications | 123817 missing removal reason | **85286** available | Includes 2283 “other device types” |
| Device dwell time + complications | 14824 missing removal reason (applied to dwell-time dataset) | **85197** available | Includes 2484 “other device types” |

These exclusions were not applied cumulatively. Each analytical component used the full initial dataset (209103 records) and only excluded cases lacking the specific variables required for that analysis. The only sequential filter was applied between the “dwell time” and “dwell time + complications” analyses, where the latter used the valid records from the former and excluded those without a documented removal reason.
