# Supplementary material for: Variability in Vascular Access Device Use, Dwell Time and Failure: Insights From Routine Nursing Documentation in a Multicentre Cohort Study
Source: J Clin Nurs. 2026 Jun 18;35(11):4828–38. doi: 10.1111/jocn.70397 (PMC13629244; doi:10.1111/jocn.70397)
Supplement: Supplementary file 13 [file JOCN-35-4828-s011.docx]

**Supplementary table 1**. the strengthening of the reporting of observational studies in epidemiology (STROBE) guidelines

|  | | | Item No. | Recommendation | | Page  No. | | | Relevant text from manuscript |
| --- | --- | --- | --- | --- | --- | --- | --- | --- | --- |
| **Title and abstract** | | | 1 | (*a*) Indicate the study’s design with a commonly used term in the title or the abstract | | 1 | | | COHORT STUDY |
|  |  |  |  | (*b*) Provide in the abstract an informative and balanced summary of what was done and what was found | | 1-2 | | | ABSTRACT |
| Introduction | | | | | | | | |  |
| Background/rationale | | | 2 | Explain the scientific background and rationale for the investigation being reported | | 3-8 | | | INTRODUCTION |
| Objectives | | | 3 | State specific objectives, including any prespecified hypotheses | | 9 | | | OBJECTIVES |
| Methods | | | | | | | | |  |
| Study design | | | 4 | Present key elements of study design early in the paper | | 9 | | | STUDY DESIGN |
| Setting | | | 5 | Describe the setting, locations, and relevant dates, including periods of recruitment, exposure, follow-up, and data collection | | 10 | | | STUDY POPULATION |
| Participants | | | 6 | (*a*) *Cohort study*—Give the eligibility criteria, and the sources and methods of selection of participants. Describe methods of follow-up  *Case-control study*—Give the eligibility criteria, and the sources and methods of case ascertainment and control selection. Give the rationale for the choice of cases and controls  *Cross-sectional study*—Give the eligibility criteria, and the sources and methods of selection of participants | | 10-11 | | | STUDY POPULATION  DATA COLLECTION |
|  |  |  |  | (*b*) *Cohort study*—For matched studies, give matching criteria and number of exposed and unexposed  *Case-control study*—For matched studies, give matching criteria and the number of controls per case | |  | | |  |
| Variables | | | 7 | Clearly define all outcomes, exposures, predictors, potential confounders, and effect modifiers. Give diagnostic criteria, if applicable | | 11 | | | DATA COLLECTION  SUPPLEMENTARY APPENDIX 2 |
| Data sources/ measurement | | | 8* | For each variable of interest, give sources of data and details of methods of assessment (measurement). Describe comparability of assessment methods if there is more than one group | | *11* | | | *DATA COLLECTION* |
| Bias | | | 9 | Describe any efforts to address potential sources of bias | | 12 | | | To ensure data quality… |
| Study size | | | 10 | Explain how the study size was arrived at | | 10 | | | … all VADs … during 2023… |
| Quantitative variables | | 11 | | Explain how quantitative variables were handled in the analyses. If applicable, describe which groupings were chosen and why | | 12 | | DATA COLLECTION | |
| Statistical methods | | 12 | | (*a*) Describe all statistical methods, including those used to control for confounding | | 12-13 | | STATISTICAL ANALISYS | |
|  |  |  |  | (*b*) Describe any methods used to examine subgroups and interactions | | 12-13 | | STATISTICAL ANALISYS | |
|  |  |  |  | (*c*) Explain how missing data were addressed | | 12-13 | | STATISTICAL ANALISYS | |
|  |  |  |  | (*d*) *Cohort study*—If applicable, explain how loss to follow-up was addressed  *Case-control study*—If applicable, explain how matching of cases and controls was addressed  *Cross-sectional study*—If applicable, describe analytical methods taking account of sampling strategy | |  | |  | |
|  |  |  |  | (*e*) Describe any sensitivity analyses | |  | |  | |
| Results | | | | | | | | | |
| Participants | | 13* | | (a) Report numbers of individuals at each stage of study—eg numbers potentially eligible, examined for eligibility, confirmed eligible, included in the study, completing follow-up, and analysed | | 13-17 | | RESULTS | |
|  |  |  |  | (b) Give reasons for non-participation at each stage | | 13 | | RESULTS | |
|  |  |  |  | (c) Consider use of a flow diagram | |  | |  | |
| Descriptive data | | 14* | | (a) Give characteristics of study participants (eg demographic, clinical, social) and information on exposures and potential confounders | | 13-14 | | HOSPITAL PARTICIPATION  PATIENT AND DEVICE CHARACTERISTICS | |
|  |  |  |  | (b) Indicate number of participants with missing data for each variable of interest | | 13-17 | | RESULTS | |
|  |  |  |  | (c) *Cohort study*—Summarise follow-up time (eg, average and total amount) | |  | |  | |
| Outcome data | | 15* | | *Cohort study*—Report numbers of outcome events or summary measures over time | | *14-16* | | *DEVICE DURATION AND COMPLICATIONS*  *DWELL TIME AND COMPLICATIONS*  *SURVIVAL ANALYSIS* | |
|  |  |  |  | *Case-control study—*Report numbers in each exposure category, or summary measures of exposure | |  | |  | |
|  |  |  |  | *Cross-sectional study—*Report numbers of outcome events or summary measures | |  | |  | |
| Main results | | 16 | | (*a*) Give unadjusted estimates and, if applicable, confounder-adjusted estimates and their precision (eg, 95% confidence interval). Make clear which confounders were adjusted for and why they were included | | 13-17 | | RESULTS | |
|  |  |  |  | (*b*) Report category boundaries when continuous variables were categorized | |  | |  | |
|  |  |  |  | (*c*) If relevant, consider translating estimates of relative risk into absolute risk for a meaningful time period | |  | | SUPPLEMENTARY TABLE 3 | |
| Other analyses | 17 | | Report other analyses done—eg analyses of subgroups and interactions, and sensitivity analyses | |  | | SUPPLEMENTARY TABLE 4  SUPPLEMENTARY TABLE 5 | | |
| Discussion | | | | | | | | | |
| Key results | 18 | | Summarise key results with reference to study objectives | | 17-25 | | DISCUSSION | | |
| Limitations | 19 | | Discuss limitations of the study, taking into account sources of potential bias or imprecision. Discuss both direction and magnitude of any potential bias | | 24 | | This study has several limitations… | | |
| Interpretation | 20 | | Give a cautious overall interpretation of results considering objectives, limitations, multiplicity of analyses, results from similar studies, and other relevant evidence | | 24-25 | | … may not reflect…  … provided by each hospital varied greatly… | | |
| Generalisability | 21 | | Discuss the generalisability (external validity) of the study results | | 25 | | … despite these limitations… | | |
| Other information | | |  | | | | | | |
| Funding | 22 | | Give the source of funding and the role of the funders for the present study and, if applicable, for the original study on which the present article is based | |  | |  | | |

*Give information separately for cases and controls in case-control studies and, if applicable, for exposed and unexposed groups in cohort and cross-sectional studies.
