# Supplementary material for: Variability in Vascular Access Device Use, Dwell Time and Failure: Insights From Routine Nursing Documentation in a Multicentre Cohort Study
Source: J Clin Nurs. 2026 Jun 18;35(11):4828–38. doi: 10.1111/jocn.70397 (PMC13629244; doi:10.1111/jocn.70397)
Supplement: Supplementary file 14 [file JOCN-35-4828-s012.docx]

**Supplementary appendix 3.** Device-specific failure rates with 95% confidence intervals

| **Device** | **Catheter failure** | **Total devices** | **Catheter failure rate** | **95% CI (Wilson)** |
| --- | --- | --- | --- | --- |
| Short PIVC | 43,908 | 77,861 | 56.4% | 56.1–56.8% |
| Micromidline | 258 | 512 | 50.4% | 46.1–54.7% |
| Midline | 248 | 800 | 31.0% | 27.9–34.3% |
| PICC | 538 | 1,636 | 32.9% | 30.7–35.2% |
| CVC | 665 | 2,194 | 30.3% | 28.4–32.3% |
